# Heavy Drinking Is Associated with Poor Blood Pressure Control in the REasons for Geographic and Racial Differences in Stroke (REGARDS) Study

**DOI:** 10.3390/ijerph8051601

**Published:** 2011-05-17

**Authors:** Suzanne E. Judd, Leslie A. McClure, Virginia J. Howard, Daniel T. Lackland, Jewell H. Halanych, Edmond K. Kabagambe

**Affiliations:** 1 Department of Biostatistics, School of Public Health, University of Alabama at Birmingham, 1665 University Boulevard, Birmingham, AL 35294, USA; E-Mail: lmcclure@uab.edu (L.A.M.); 2 Department of Epidemiology, School of Public Health, University of Alabama at Birmingham, Birmingham, AL 35294, USA; E-Mails: vjhoward@uab.edu (V.J.H.); edmondk@uab.edu (E.K.K.); 3 Department of Neurosciences, Medical University of South Carolina, Charleston, SC 29425, USA; E-Mail: lackland@musc.edu; 4 Department of Medicine, University of Alabama at Birmingham, Birmingham, AL 35294, USA; E-Mail: jhalanych@uab.edu

**Keywords:** diabetes, race, alcohol, blood pressure, hypertension

## Abstract

Alcohol intake has been shown to have a J-shaped association with blood pressure (BP). However, this association has not been examined in mixed race populations or in people with diabetes where tighter blood pressure control is recommended. Participants in the REGARDS study who were 45 years or older (n = 30,239) were included. Medical history (including self-reported alcohol intake) was collected by telephone while blood collection and clinical measurements were done during an in-home visit. We defined diabetes as use of medications and/or fasting glucose ≥ 126 mg/dL and hypertension as use of blood pressure lowering medications and/or BP ≥ 140/90 mmHg or BP ≥ 130/80 mmHg in people with diabetes. After adjustment for confounders, heavy drinking was associated with an increased odds of hypertension (OR = 1.59; 95% CI = 1.37, 1.87). Diabetes and gender significantly modified (interaction *P* < 0.05 for both) the association between alcohol use and hypertension, although heavy drinking remained associated with increased odds of hypertension in sub-group analyses. We did not observe the previously described J-shaped relationship in any sub-group except white females. These data suggest heavy alcohol consumption is associated with poor BP control and that heavy drinkers may want to consider limiting alcohol intake in order to manage hypertension.

## 1. Introduction

Excess consumption of alcohol has been consistently shown to increase blood pressure [1,2] and is a risk factor for development of hypertension [3]. In clinical trials, reduction in alcohol intake, at any level, consistently reduces blood pressure [2]. However, in cohort studies, moderate consumption of alcohol is associated with decreased incidence of hypertension [4] and improved cardiovascular biomarkers such as higher HDL-cholesterol [5] and lower C-reactive protein (CRP) [6,7]. In some studies, even moderate alcohol consumption has been associated with detrimental effects on the cardiovascular system [4,8]. The inconsistencies between studies and the well-documented J-shaped relationship between alcohol and blood pressure have complicated recommendations about the potential benefit of moderate alcohol consumption [1].

One reason for the inconsistencies among research studies is that in defining alcohol categories many studies fail to address the sex-specific cut-points for alcohol intake that have been recommended by the National Institute of Alcohol Abuse and Alcoholism (NIAAA) [9]. Failure to use these lower cut-points for women effectively reduces the sample size for the referent group (moderate consumption) and misclassifies some heavy drinkers as moderate drinkers. In addition, the association of alcohol and hypertension may vary by history of diabetes and race, thus potentially accounting for inconsistencies across studies [10,11]. Finally, most studies had few minority groups, who typically report higher rates of abstaining from alcohol [4,12], making it difficult to disentangle alcohol-race interaction from variability attributable to small sample sizes in subgroups.

The REasons for Geographic And Racial Differences in Stroke (REGARDS) national cohort, with 30,239 white and black participants, provides a unique opportunity to test for potential interactions between alcohol intake, race, and their associations with hypertension. Moreover, this study has sufficient statistical power to further test whether covariates such as diabetes modify the effect of alcohol on blood pressure control and/or hypertension.

## 2. Experimental Section

### 2.1. Study Population

The REGARDS study comprises a cohort residing in the United States designed to prospectively examine racial and regional disparities in stroke risk and mortality (www.regardsstudy.org). Detailed design and methods for the REGARDS study have been described elsewhere [13]. Black and white participants, aged 45 and older, oversampled from the southeastern stroke belt and buckle, were recruited in 2003–2007 from commercially available lists, and screened during a phone interview to determine eligibility. REGARDS is similar, in terms of age and socioeconomic status, to the national population if compared to the Behavioral Risk Factor Surveillance Study from 2005 and the Census in 2000. The Stroke Belt is a well characterized region in the United States with excessively high stroke mortality. North Carolina, South Carolina, Georgia, Alabama, Mississippi, Tennessee, Arkansas and Louisiana are all included in the Stroke Belt. The Stroke Belt “buckle” represents a region within the Stroke Belt of even higher stroke mortality and includes counties along the coastal plain of North and South Carolina and Georgia. Following verbal consent, using a computer-assisted telephone interview (CATI), trained interviewers obtained demographic information and medical history. A physical examination including blood pressure measurements, blood and urine samples, and an electrocardiogram (ECG) was conducted in-person 3–4 weeks after the telephone interview, and written informed consent was obtained. All involved Institutional Review Boards approved the study methods. The telephone response rate was 33% and cooperation rate was 49%, similar to other cohort studies [14].

### 2.2. Alcohol Use

Alcohol intake, the main exposure, was assessed with the following questions during the telephone interview: “Do you presently drink alcoholic beverages, including beer, wine, and other drinks made with hard liquor, even occasionally?” If the answer was yes, the participant was then asked “How many alcoholic beverages do you presently drink?” Presently was not specifically defined in the questionnaire. We defined alcohol use according to the NIAAA classifications *i.e.*, moderate (1 drink per day for women or 2 drinks per day for men) and heavy alcohol use (greater than 1 drink per day for women and 2 drinks per day for men). Compared to life-long abstainers, former drinkers (could include “sick quitters”) may be at higher risk for hypertension and poor health in general [15]. Thus, we initially examined former drinkers and never drinkers as separate categories in models that tested the association between alcohol consumption and blood pressure or hypertension. In all models, we found no significant difference between former drinkers and never drinkers. Therefore the self-reported alcohol use categories in the current analyses are non-drinkers, moderate drinkers, and heavy drinkers.

### 2.3. Hypertension and Blood Pressure

We defined hypertension as use of medications to lower blood pressure (from CATI), or blood pressure at or above 140/90 mmHg in people without diabetes and at or above 130/80 mmHg in people with diabetes [1]. History of hypertension was queried in the CATI telephone interview. Self-reported hypertension and use of antihypertensive medications were ascertained using the following questions: “Has a doctor or other health professional ever told you that you have high blood pressure?” and “Are you NOW taking any medicine for high blood pressure?”

We used two outcome variables, systolic blood pressure (SBP) as a continuous variable and hypertension as a binary variable. During the in-home visit, trained, certified health professionals measured blood pressure using aneroid sphygmomanometers with the appropriate cuff size. Blood pressure quality control is monitored by central examination of digit preference, and retraining of technicians takes place if necessary. The participant was seated in a chair with both feet on the floor for 3 minutes. Blood pressures were repeated twice and averaged for the analyses.

### 2.4. Diabetes and Blood Sugar

Diabetes was defined as a fasting glucose level ≥ 126 mg/dL, non-fasting glucose ≥ 200 mg/dL, or self-reported medication use for glucose control. Self-reported diabetes and use of blood sugar lowering medications were ascertained using the following questions during the CATI telephone interview: “Has a doctor or other health professional ever told you that you had diabetes or high blood sugar?” followed by “Did you have diabetes only while you were pregnant?” and “Are you taking medicine for diabetes?” Participants were asked to fast prior to in home visit. A trained health professional collected blood samples and all samples were sent to the University of Vermont for processing. Glucose was measured in serum using a colorimetric reflectance spectrophotometry on the Ortho Vitros 950 IRC Clinical Analyzer (Johnson & Johnson Clinical Diagnostics, Rochester, NY, USA) with a C.V. of 1%.

### 2.5. Covariates

Comorbid medical conditions of interest *i.e.*, cardiovascular (CVD), and stroke were determined by objective measures or self report. Prevalent CVD was supported by evidence of myocardial infarction on ECG. Smoking was categorized into strata of current, former, and never smoker. Body mass index (BMI) was calculated from height and weight measured during the in-home visit. Physical activity level was defined by response to the CATI question “How many times per week do you engage in intense physical activity, enough to work up a sweat?”, categorized as a healthy level if 4 or more times per week, light if 1–3 times a week or none. Socioeconomic variables such as employment status, income, and education, were self-reported during the CATI interview.

### 2.6. Statistical Methods

We used multivariable logistic regression models (SAS version 9.2, Cary, NC, USA) to calculate odds ratios and corresponding 95% confidence intervals for the association between alcohol consumption and hypertension. Covariates of interest were geographic region (stroke belt, stroke buckle, non-belt), employment status, income, education, sex, smoking, physical activity, and history of diabetes, CVD, or stroke. We used Pearson’s chi-square test to obtain a *P*-value to compare frequency distributions of alcohol use patterns. Potential confounders such as C-reactive protein, BMI, age, HDL- and LDL-cholesterol were modeled as continuous covariates after appropriate transformations were made to account for data structure.

We hypothesized that diabetes and race could modify the relation between alcohol use and hypertension. Thus, we tested two interactions *i.e.*, alcohol use and diabetes and alcohol use and race for their effect on the association between alcohol use and hypertension. If the association was significant we performed analyses stratified by these variables. In the screening models, variables with a *P-*value for interaction < 0.10 were considered significant. Potential confounders described above were sequentially added to models that already contained the interaction terms for history of diabetes, and odds ratios and 95% CI were estimated. We used backwards elimination to determine significant factors in the models. Confounders with *P*-values < 0.20 were retained in models.

For adjusted mean blood pressure analysis, we used linear regression and employed similar techniques for model building as those described for logistic models. Reported mean SBPs are adjusted for covariates in the models. After all models were built, we conducted sensitivity analyses to determine the impact of past drinking. To achieve this, we split the group reporting no current alcohol consumption into past drinkers and never drinkers.

## 3. Results and Discussion

The total REGARDS cohort contains 30,239 individuals. We excluded individuals with missing data on blood pressure measurements (n = 95), history of hypertension/medication use (n = 112), alcohol use (n = 593) and fasting glucose measures (n = 949). This left an analysis population of 28,490 participants.

Among the 28,350 men and women included in the final analyses, 63% reported not consuming alcohol, while 33% were moderate drinkers and 4% were heavy drinkers. The prevalence (Table 1) of heavy alcohol consumption was highest in whites (76%), males (54%) and among individuals with higher income (11% of heavy drinkers reported making less than $20,000 and 25% of heavy drinkers reported making more than $75,000). Smoking was more common in heavy drinkers (29%) than never drinkers (12%) as was physical activity (37% of heavy drinkers reported exercising more than 4 or more times per week compared to 27% of non-drinkers).

The mean age (Table 1) of non-drinkers was slightly higher than that of heavy drinkers (65.5 *vs.* 63.4, *P* < 0.001). As expected, the median HDL-cholesterol concentration was lowest (48 mg/dL) in non-drinkers and higher in moderate (50 mg/dL) and heavy drinkers (58 mg/dL), despite the lower proportion of women among the latter group. BMI was lower in heavy drinkers compared with non-drinkers (27.2 *vs.* 29.9, *P* < 0.001). Additionally, the prevalence of diabetes was lower among heavy drinkers (10%) than moderate drinkers (15%), while non-drinkers had the highest prevalence of diabetes (26%).

We examined in a stepwise fashion, the association between alcohol use category and hypertension (Table 2). Moderate drinkers were less likely to be have hypertension in age, race and sex adjusted models (OR = 0.79; 95% CI = 0.75, 0.84), however, this association was null in fully adjusted models (OR=1.03; 95% CI = 0.97, 1.95). Although heavy drinking was associated with a lower odds of hypertension in crude models, in fully adjusted models heavy drinking was associated with higher odds of hypertension.

In order to examine our *a priori* hypothesis that the association of alcohol intake with hypertension might vary across subgroups, we stratified on race, gender, and diabetes. In general, age-adjusted models showed that moderate alcohol consumption was associated with lower odds of hypertension. Women were the only subgroup in which this association held up after adjustment for all other risk factors. In contrast, heavy drinking was generally associated with increased odds of hypertension across subgroups after adjusting for other covariates. Since it is possible that the interaction with diabetes could have been driven by the fact that the definition of hypertension among people with diabetes was different from that used among those without diabetes, we conducted sensitivity analyses that used the same definition for all study participants. In these analyses the interaction was no longer present (*P* for interaction = 0.40), and the strength and significance of association was attenuated in heavy drinkers with diabetes (OR = 1.55; 95% CI = 0.94, 2.60); however, the direction of association remained the same.

**Figure 2.**
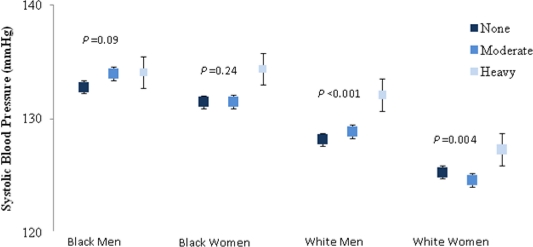
Adjusted mean systolic blood pressure (SBP) by alcohol use pattern (defined according to National Institute on Alcohol Abuse and Alcoholism (NIAAA) guidelines) among The REasons for Geographic And Racial Differences in Stroke (REGARDS) Study participants. The association of SBP with alcohol use differed by race (*P* = 0.02) and gender (*P* < 0.001). Models adjust for age, region of residence, income, education, smoking status, physical activity, BMI, HDL, LDL, CRP, and history of stroke, diabetes, and heart disease. We defined alcohol use: none, moderate (1 drink per day for women or 2 drinks per day for men) and heavy alcohol use (greater than 1 drink per day for women and 2 drinks per day for men).

In analyses of adjusted mean SBP in each of the three alcohol use categories stratified by race and gender grouping (Figure 2). The association differed by race (*P* for interaction = 0.02) and by gender (*P* for interaction < 0.001) and by gender-race grouping (*P* for interaction < 0.001). In blacks (regardless of gender), there was not a statistically significant association between alcohol use and SBP, although there was a trend for increasing SBP from non-drinkers to heavy drinkers in analyses that controlled for covariates. In whites (regardless of gender), adjusted mean SBP was higher (*P* < 0.01) in heavy drinkers than in moderate drinkers and non-drinkers.

## 4. Conclusions

In a large population of blacks and whites in a national cohort, we found that heavy drinking is significantly associated with increased SBP and a higher prevalence of hypertension. The association between alcohol use and hypertension was stronger among individuals who have diabetes than those without diabetes in models that controlled for several known hypertension risk factors including cigarette smoking and race.

Maintaining blood pressure control is important in people with diabetes to prevent future co-morbidities. There has been limited clinical evidence to suggest that alcohol should be avoided in people with diabetes, and, in fact, some suggest moderate alcohol intake improves insulin sensitivity [16]. Current recommendations for alcohol use among people with diabetes focus on avoiding hypoglycemia, but it may be prudent to also consider co-morbidities such as hypertension when making recommendations. The Diabetes Prevention Program demonstrated that control of diet and lifestyle can improve blood sugar control [17]. As new recommendations strongly advise better management of blood pressure in persons with diabetes to reduce their cardiovascular risk, and in this study heavy alcohol use was strongly associated with hypertension in people with diabetes, alcohol consumption may be one of the lifestyle factors to modify among people with diabetes.

Due to the potential adverse effects (addiction, liver damage, and social consequences), recommendations regarding alcohol consumption solely for CVD risk reduction are not well defined. Current American Heart Association recommendations indicate “if one drinks alcohol, to drink in moderation (no more than 2 drinks per day in men and 1 drink per day in women)” [18]. However, it is unclear if this broad recommendation is applicable to all segments of the population including all races. The Atherosclerosis Risk in Communities (ARIC) study (ages 45–64 at baseline) demonstrated that moderate drinkers who were black but not white were at increased risk for hypertension [4]. This same group went on to demonstrate that this same group of black participants who reported drinking at any level were at slightly higher risk of coronary heart disease [10]. In contrast, in younger individuals in the Coronary Artery Risk Development in Young Adults (CARDIA) study, there was not a differential association of alcohol with incident hypertension [12]. Our findings are similar in that there was no association of prevalent hypertension with alcohol in blacks. To our knowledge, no study has yet examined whether there is a differential effect of alcohol in people living with diabetes on the prevalence of hypertension, and whether these associations may differ by race. Our data suggest considering both race and history of diabetes separately when examining alcohol use as a risk factor for hypertension. This is particularly relevant given the diverse alcohol consumption practices in the different populations around the world.

In the initial analyses, we were concerned about the robustness of the models in two ways. First, we were concerned that those in the non-drinker alcohol use group might be in that group because they were “sick quitters” [19]. We therefore performed analyses in which we tested whether past drinkers and never drinkers were significantly different with regard to hypertension or SBP. In all models, past drinkers were not significantly different from never drinkers; therefore, models contain past drinkers and never drinkers in the non-drinker category. Previous studies have shown a protective effect of moderate alcohol consumption. It is possible due to the cross-sectional nature of this analysis, participants with established hypertension had already limited alcohol consumption in order to help control blood pressure. Even with the sensitivity analysis described above, we are unable to establish causality between alcohol intake and hypertension due to the cross-sectional nature of the study.

It is possible that dietary intakes of specific nutrients such as sodium and/or saturated fat may be different across alcohol use categories. One major limitation of the study is that we did not have dietary information on the whole cohort of interest and so we were not able to examine this association. We did however have diet data on a subset of the cohort. Addition of dietary factors into the models did not alter the odds ratios in a way that would change the interpretation of the results. The confidence intervals were slightly wider and some overlapped with the null value due to the decrease in the sample from 28,490 to 20,433 when dietary factors were added to the model. Therefore, we used the whole cohort for the analysis so we would be powered to detect the interactions of interest.

We were limited in that we have only a one time measure of blood pressure and alcohol intake, and were only able to examine the cross-sectional association of alcohol with blood pressure. Since the study was cross-sectional in nature, this creates the potential for reverse causation where the exposure of interest is actually driven by the outcome. Future studies could examine alcohol intake and risk of incident hypertension and sequela of uncontrolled hypertension such as stroke or heart disease. Furthermore, we were not able to control for the dose and type of blood pressure medication the study participants may have used. We were also limited in that we had only self-report of alcohol intake from the telephone survey. We obtained only average daily alcohol consumption and do not have information on patterns of alcohol consumption. In addition, we did not query as to type of alcohol consumed. As alcohol content of various drinks may vary, this could be a limitation of the study. However, we have a large population with substantial numbers of both white and black participants to observe whether the association of alcohol with blood pressure is similar to other studies.

These data suggest that heavy alcohol consumption is associated with poor BP control in people regardless of sub-group. Since the blood pressure management can be complicated, encouraging people who drink heavily to reduce alcohol consumption may provide a benefit for blood pressure management. Future studies examining blood pressure control in people with diabetes and how that is associated with race and other dietary factors seem warranted.

## Figures and Tables

**Figure 1.**
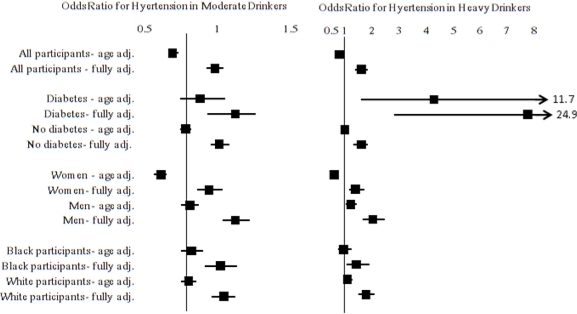
Forest plot exploring interactions between diabetes, race, and sex and alcohol intake on the odds of having hypertension in the Reasons for Geographic And Racial Differences in Stroke (REGARDS) study participants. Non-drinkers are the referent group. Diabetes was a significant effect modifier (*P* for interaction = 0.02), while race was marginally non-significant (*P* for interaction = 0.10) and gender was highly significant (*P* for interaction < 0.001). The fully adjusted model controlled for age, race, sex, region of residence, income, education, smoking status, physical activity, BMI, HDL, LDL, CRP, diabetes, and history of stroke, and heart disease. Values in the figure are odds ratios and corresponding 95% CIs.

**Table 1.** Demographic, socioeconomic, and lifestyle characteristics of participants in the REasons for Geographic And Racial Differences in Stroke (REGARDS) Study (2003–2007) by alcohol category (values presented are means ± standard deviations or medians with 25th and 75th percentile ranges if not normally distributed). We defined alcohol use: none, moderate (1 drink per day for women or 2 drinks per day for men) and heavy alcohol use (greater than 1 drink per day for women and 2 drinks per day for men).

	**Alcohol use category**			
	**Non-drinkers**	**Moderate**	**Heavy**	

Total	1,7815 (63%)	9,531 (33%)	1,144 (4%)	*p†*
White race	9,383 (53%)	6,580 (69%)	865 (76%)	<0.001
Male	6,756 (38%)	5,450 (57%)	622 (54%)	<0.001
Residing in stroke belt	10,605 (59%)	4,652 (49%)	600 (52%)	<0.001
Current smoking	2,217 (12%)	1,555 (16%)	337 (29%)	<0.001
History of disease				
Hypertension	11,084 (62%)	5,056 (53%)	654 (57%)	<0.001
Stroke	1,323 (7%)	434 (5%)	48 (4%)	<0.001
Heart disease	4,130 (23%)	2,039 (21%)	203 (18%)	<0.001
Diabetes	4,664 (26%)	1,436 (15%)	117 (10%)	<0.001
Income category				
$20K	3,988 (22%)	995 (10%)	126 (11%)	
$20K–$34K	4,699 (26%)	1,965 (21%)	227 (20%)	
$35K–$74K	4,889 (27%)	3,212 (34%)	372 (33%)	
$75+	1,822 (10%)	2,442 (26%)	286 (25%)	
Did not report	2,417 (14%)	917 (10%)	133 (12%)	<0.001
Education				
<High school	2,839 (16%)	614 (6%)	90 (8%)	
High school	5,197 (29%)	1,905 (20%)	251 (22%)	
Some college	4,764 (27%)	2,547 (27%)	298 (26%)	
College+	4,998 (28%)	4,463 (47%)	505 (44%)	<0.001
Physical activity				
None	6,558 (37%)	2,708 (28%)	339 (30%)	
1–3 times per week	6,091 (34%)	3,677 (39%)	364 (32%)	
4+ times per week	4,884 (27%)	3,033 (32%)	419 (37%)	<0.001
Mean or medians				
Age (years)	65.5 ± 9.5	63.8 ± 9.3	63.4 ± 8.9	<0.001
SBP (mmHg)	128.2 ± 16.8	126.3 ± 16.5	128.1 ± 16	<0.001
DBP (mmHg)	76.5 ± 9.9	76.5 ± 9.5	77.1 ± 9.4	0.12
HDL (mg/dL)	48 [40, 59]	50 [41,62]	58 [47, 72]	<0.001
LDL (mg/dL)	111 [89, 136]	111 [90, 34]	109 [90, 133]	<0.001
CRP (mg/L)	2.5 [1.0, 5.6]	1.9 [0.9, 4.3]	2.0 [0.8, 4.6]	<0.001
BMI (kg/m^2^)	29.9 ± 6.5	28.5 ± 5.6	27.2 ± 5.2	<0.001

**Table 2.** Odds ratios (95% CI) for the association between alcohol intake and hypertension in the REasons for Geographic And Racial Differences in Stroke (REGARDS) Study (2003–2007).

	**Alcohol use category**		
	**Non-drinkers (n = 17,815)**	**Moderate drinkers (n = 9,531)**	**Heavy drinkers (n = 1,144)**
All participants—crude model	1.0	0.66 (0.63, 0.69)	0.78 (0.69, 0.88)
All participants—age-adjusted model	1.0	0.69 (0.66, 0.73)	0.84 (0.74, 0.95)
All participants—age-, sex-, race-adjusted model	1.0	0.79 (0.75, 0.84)	1.04 (0.91, 1.18)
All participants—model adjusted for age, sex, race, region of residence, income, education, smoking status, physical activity, HDL, LDL, CRP, BMI, history of diabetes, and history of stroke, and heart disease.	1.0	1.03 (0.97, 1.95)	1.68 (1.45, 1.95)
